# A retrospective review of screening for HIV, syphilis and anaemia on first antenatal care visits at a midwife-run obstetric unit in South Africa

**DOI:** 10.4102/safp.v68i1.6261

**Published:** 2026-04-09

**Authors:** Keshena Naidoo, Monjural Hoque, Maariyah Hoque, Somaya Buckus

**Affiliations:** 1 School of Medicine, Department of Family Medicine, Faculty of Health Sciences, Nelson Mandela University, Gqerberha, South Africa; 2 Discipline of Family Medicine, College of Health Sciences, University of KwaZulu-Natal, Durban, South Africa; 3 KwaDabeka Community Health Centre, eThekwini Health District, KwaZulu-Natal Department of Health, Clermont, South Africa; 4 South African College of Applied Psychology, Faculty of Applied Psychology, Durban, South Africa

**Keywords:** antenatal care, maternal health, pregnant women, primary health care, women’s health

## Abstract

**Background:**

Early enrolment for antenatal care (ANC) is an important strategy in improving perinatal and maternal outcomes. Treatable conditions in pregnancy, such as anaemia, human immunodeficiency virus (HIV), and syphilis can be identified and treated in the early antenatal period, thereby improving maternal and foetal outcomes. However, late enrolment for ANC is a concerning problem, especially in high HIV-prevalence settings, such as South Africa. The aim of this study was to evaluate screening of pregnant women fro HIV, syphilis and anaemia on the first antenatal visit at a midwife-run obstetric unit in KwaZulu-Natal, South Africa.

**Methods:**

This audit of maternity case records at a primary health care facility in South Africa focuses on the screening for treatable conditions (i.e. HIV, syphilis and anaemia) among women presenting for their first antenatal visit.

**Results:**

Data were extracted from the files of 400 (87.7%) of the 456 women who enroled for ANC between July and December 2023. There was good coverage for screening of HIV, syphilis and anaemia. The prevalence of anaemia among women enrolling for ANC was 25% (*n* = 99), 40.8% (*n* = 100) of participants were HIV positive (*n* = 100) and 2.5% (*n* = 10) tested positive for syphilis. Of concern, less than 20% (*n* = 72) enrolled for ANC in the first trimester, thereby limiting the effectiveness of ANC interventions.

**Conclusion:**

The low uptake of early ANC services is of concern, especially in high HIV-prevalence settings.

**Contribution:**

Training of primary health care providers in point-of-care ultrasounds may improve community awareness of the benefits of early ANC. Further investigation is required into the knowledge and perceptions of women regarding ANC services.

## Introduction

Early enrolment for antenatal care (ANC) is an important strategy in attaining Sustainable Development Goal 3.1 of reducing maternal mortality rates to < 70 per 100 000 livebirths by 2030.^1,2^ Sub-Saharan Africa (SSA) has the highest estimated maternal mortality ratio in the world, 542 reported deaths per 100 000 live births in 2017.^3,4^ Preventable causes of maternal mortality in SSA such as anaemia, infections and hypertensive disorders can be identified and treated in the early antenatal period, thereby improving maternal and foetal outcomes.^5^ The World Health Organization (WHO) recommends that the first ANC visit take place within the first trimester (gestational age [GA] of < 12 weeks) with an additional seven visits.^6^ Antenatal care when provided in the first trimester, or no later than 16 weeks of pregnancy, enables timely intervention for pregnancy-related complications and can potentially prevent maternal and perinatal deaths from avoidable causes.^7,8,9^

Despite the evidence on the benefits of early initiation of ANC, the uptake of early ANC in SSA remains poor.^10^ A recent systematic review reported that the proportion of pregnant women in SSA who initiated ANC early (less than 12 weeks of gestation) ranged from 14.5% in Mozambique to 47.3% in Cameroon to 68.6% in Liberia, with an overall rate of 38.0% (95% CI: 37.8–38.2).^11,12^ Women with secondary or higher education, higher income households, close proximity to health facilities and exposure to media were more likely to initiate ANC early.^11^ Past obstetric history is also a strong predictor of early initiation of ANC as women with previous abortions, caesarean sections and pregnancy-related complications are more likely to present early.^13^ Late presentation for ANC was more likely with women in rural areas and those who had unplanned pregnancies.^11,14^

Apart from social and obstetric factors, the perceptions of women regarding ANC is an important determinant of the timing of ANC.^5^ Most women consider pregnancy as a normal part of life and not a health issue. There are also misconceptions about early ANC as many pregnant women perceive that early ANC visits are only for those with severe symptoms, such as vomiting, backache and headache and those with HIV.^13^ Previous positive pregnancy outcomes also undermine the value of early ANC in health facilities.^5^ Many pregnant women are often reportedly reluctant to start ANC because of long waiting times at clinics, overcrowded conditions and negative experiences of healthcare providers.^5,15^

South Africa, a middle-income country in SSA, implemented the WHO ANC recommendations through the Basic Antenatal Care (BANC) policy in 2017 with results similar to other SSA countries.^16^ Despite the availability of free ANC, only 69.6% of pregnant women enroled within the first 20 weeks of gestation, ranging from 64.7% in KwaZulu-Natal to 86.1% in the Northern Cape.^17^ Recommendations from the recent Saving Mothers report to prevent maternal deaths included early identification of pre-existing medical conditions and hypertensive disorders in pregnancy.^18^ When BANC was implemented early, there was a significant improvement in the detection and management of hypertensive disorders in pregnancy.^16^ However, an audit of ANC records in KwaZulu-Natal identified several gaps in the implementation of BANC on the first ANC visit.^19^ This study evaluates the prevalence of late booking for ANC and audits the adherence to screening guidelines among pregnant women on their first visit at a primary health facility in KwaZulu-Natal, South Africa.

## Research methods and design

### Study design

This was a quantitative descriptive study. A retrospective record review was conducted of maternal health records of women who enroled for ANC between July and December 2023 at a primary health care facility in South Africa.

### Study setting

The study was conducted at a peri-urban midwife-run obstetric unit (MOU) on the western border of the Durban (eThekwini) Metropolitan city in KwaZulu-Natal, South Africa.

The health facility serves a low socio-economic community with a high level of unemployment. The facility reports approximately over 1500 pregnant women who attend the MOU for ANC per year.

Clinical services are provided by professional nurses and district medical officers. Women with high-risk pregnancies and who require operative delivery are referred to a regional hospital.

### Study population

**Inclusion criteria:** Pregnant women presenting between July 2023 and December 2023 to the antenatal clinic for their first visit were included.

**Exclusion criteria:** Pregnant women who had come for follow-up visits and who had started ANC at another facility were excluded.

### Data collection

Trained research assistants extracted data from the clinic registers and maternity case records (MCR) using a validated data collection tool. Data were extracted on socio-demographic characteristics, medical and obstetric history and GA at first antenatal clinic visit. The records were audited to assess adherence to the standard of care on first visit that includes estimation of GA, screening and treatment for anaemia, HIV and syphilis. Ultrasound services are not available at the facility. Estimation of the GA was calculated using self-reported last menstrual period or the symphysis-fundal height (SFH).^2,20^ Anaemia was diagnosed using a hand-held haemoglobinometer. Healthcare providers used a point-of-care test to screen for syphilis. A positive syphilis test indicates either prior syphilis or current syphilis. All pregnant women with a positive syphilis test received one dose of penicillin and had blood sent to a laboratory for another syphilis test. Early booking was defined as enroling for ANC before 20 weeks of gestation.

### Data analysis

Data were captured on a Microsoft Excel spreadsheet and imported into the statistical software programme, SPSS 27.0 (SPSS Inc., Chicago, IL, USA), for further analysis. Both continuous and categorical variables were described using descriptive statistics. The outcome of interest was late booking (first visit after 20 weeks of GA). Cross-table analysis using Pearson’s chi-square (X^2^) and *p* values were applied to evaluate the effect of variables on the outcome. Binary logistic (backward) regression analysis was performed on the relevant variable (*p* < 0.05).

### Ethical considerations

Ethical clearance to conduct this study was obtained from the Umgungundlovu Health Ethics Research Board (No. UHERB 015/2020). Gatekeeper permission was obtained from the facility management prior to data collection. Data were captured from clinical records; no direct involvement with participants occurred. Participants’ names were anonymised and kept confidential. Data were stored on a password-protected laptop. Data will be destroyed after 5 years.

## Results

### Characteristics of participants

A total of 456 pregnant women enroled for ANC between July and December 2023. The sample size was 400 (87.7%) as not all records were complete. The socio-demographic profile of participants who booked during the study period is presented in Table 1.

**TABLE 1:** Socio-demographic characteristics and gestational age at booking of women enrolling for antenatal care.

Variable	*n*	%	Mean	s.d.
**Age in years**	-	-	26.5	± 6.8
< 20	99	25.0	-	-
20–30	174	43.8	-	-
31–40	116	28.7	-	-
> 40	11	2.5	-	-
**Parity (number of deliveries)**	-	-	1.23	1.2
0	129	32.3	-	-
1–2	220	55.0	-	-
3–4	47	11.8	-	-
> 5	4	1.0	-	-
**Marital status**				
Single	335	83.5	-	-
Married	40	10.0	-	-
Divorce or widowed	25	2.5	-	-
**Education**				
Primary school or less	23	5.7	-	-
Completed high school	259	64.7	-	-
Attended tertiary education	118	29.6	-	-
**Employment**				
Employed	90	22.5	-	-
Unemployed	310	77.5	-	-
**Gestational age at first antenatal care visit**	-	-	18.7	6.1
< 13 weeks (first trimester)	72	18.0	-	-
13–26 weeks (second trimester)	292	73.0	-	-
> 26 weeks (third trimester)	36	9.0	-	-
**Gestational age (weeks) at booking for antenatal care**				
≤ 20	80	20.0	-	-
> 20	320	80.0	-	-

s.d., standard deviation; GA, gestational age.

The ages of women at the first antenatal visit ranged from 15–45 years, with a mean age of 26.5 years (s.d. = 6.8). The majority of women were between 20–40 years old (72.5%), and 11 (2.5%) women were older than 40 years. However, 99 (25%) women were teenagers. The number of previous deliveries ranged from 0–7 deliveries, with a mean parity of 1.23 deliveries (s.d. = 1.2). More than half (55.0%) had a parity between 1 and 2. A few (1.0%) had grand multiparity (parity 5 or more). The majority of the women were unmarried (83.5%), had completed high school education (64.7%) and were unemployed (77.5%).

The mean GA at the first ANC visit was 18.7 (s.d. = 6.1) weeks, with a minimum of 8 weeks and a maximum of 32 weeks. However, the majority of pregnant women (73.7%) initiated ANC in the second trimester (between 13 and 26 weeks) of pregnancy, and most (*n* = 321; 80.0%) of the women presented after 20 weeks of GA.

### Prevalence of treatable conditions

The outcomes of the screening tests for HIV, syphilis and anaemia at first ANC visit are presented in Table 2.

**TABLE 2:** Results of screening tests for HIV, syphilis, and anaemia at first antenatal care visit (*N* = 400).

Treatable condition screened and treated	*n*	%
**HIV screening**		
• Known positive before first visit	143	35.8
• Screened at first visit (negative or unknown status at booking)	253	63.3
Tested positive at first visit	20	-
Tested negative at first visit	233	-
• Declined to test (*n* = 4 [1.0%])	4	1.0
**HIV Treatment (*N* = 163)**		
• HIV-positive pregnant women at first visit	163	100
• HIV-positive women started on treatment	151	98.7
**Syphilis screening**		
• Tested negative at first visit	390	97.5
• Tested positive at first visit	10	2.5
• Treatment of syphilis positive cases (*n* = 10)	9	90.0
**Screening for anaemia**		
• Hb > 11 g/dL	301	75.0
• 10–10.9 g/dL (mild anaemia)	64	16.3
• 8–9.9 g/dL (moderate anaemia)	29	7.2
• < 8 g/dL (severe anaemia)	6	1.5

Overall, during enrolment for ANC, 143 women were found to be HIV positive; all of whom were on antiretroviral therapy (ART). Screening for HIV was offered to all women who did not know their status or who previously tested negative (*n* = 257), of whom four (1%) declined to test. Twenty pregnant women were newly diagnosed as HIV positive at their first visit to ANC. A total of 163 pregnant women were identified as HIV positive, of which 98.7% (*n* = 151) were on treatment by the end of the first visit. All women were screened for syphilis and 10 women tested positive for syphilis (2.5% prevalence rate). Nine women (90%) received treatment for syphilis. The prevalence of anaemia was 25% (*n* = 99). Mild anaemia was the most common form of anaemia (16.3%; *n* = 64), followed by moderate anaemia (*n* = 29; 7.2%) and severe anaemia (*n* = 6; 1.5%).

### Association between late antenatal care booking and maternal characteristics

The characteristics of women who enroled late (> 20 weeks of GA) were compared to those who enroled before 20 weeks of GA in Table 3 to identify possible predictive factors for late booking.

**TABLE 3:** Factors associated with late antenatal care booking.

Variable	Late booking *N* = 320		Early booking *N* = 80		*X*^2^ value	*P*
	*n*	%	*n*	%		
**Age group (years)**						
< 20	82	83.5	17	16.5	1.242	0.743
20–30	141	80.8	33	19.2	-	-
31–40	89	76.9	27	23.1	-	-
> 40	8	77.8	3	22.2	-	-
**Marital status**						
Single	269	80.3	66	19.7	0.060	0.970
Married	31	78.3	9	21.7	-	-
Divorced or widowed	20	80.0	5	20.0	-	-
**Education**						
Primary or nil	17	73.9	6	26.1	1.242	0.537
Completed secondary	210	81.2	49	18.8	-	-
Tertiary	93	79.2	25	20.8	-	-
**Employment status**						
Employed	68	75.9	22	24.1	1.262	0.261
Unemployed	252	81.5	58	18.5	-	-
**Parity (number of deliveries)**						
0	110	85.2	19	14.8	2.964	0.397
1–2	173	78.6	47	21.4	-	-
3–4	35	75.0	12	25.0	-	-
≥ 5	3	75.0	1	25.0	-	-

There were no significant associations noted regarding age, marital status, education, employment of parity and late initiation of women in ANC (> 20 weeks of gestation).

## Discussion

The services provided at the study facility to pregnant women on the first antenatal visit met expected standards. Almost all women were screened and managed for treatable conditions such as anaemia, HIV and syphilis on their first visit. However, in the majority of women, these conditions were detected after their first trimesters. Only 18% of pregnant women started ANC in the first trimester (12 weeks of gestation) and 20% booked within 20 weeks of gestation. The prevalence of late booking in this study was much higher than that reported in other studies in South Africa and SSA.^21^ No association could be found between the timing of booking and the age, marital status, education, employment status or parity. This differs from other studies where higher levels of education, partner support, office employment and null parity were all found associated with early booking.^11^ One possible factor for late enrolment for ANC could be the rural residence of women who used ANC services. Studies in SSA report that women in rural locations were more likely to book late.^14^ Also, socio-cultural practices such as the use of traditional health practitioners and traditional medicines may delay attendance at antenatal clinics by pregnant women.^22^

The GA of pregnant women when initiating ANC was estimated using the SFH or the last normal menstrual period. At the time of the study, there were no ultrasound services at primary care clinics. Hence, women could only access an ultrasound in the private sector or at regional hospitals. The WHO recommends that all pregnant women have one antenatal ultrasound before 24 weeks of pregnancy.^23^ Early ultrasound provides accurate GA and important information about pregnancy that could encourage women to access ANC services earlier. However, in resource-constrained settings in SSA, there is a scarcity of trained ultrasound technicians and limited ultrasound services. Several studies have investigated how training of primary care providers in point-of-care ultrasound (POCUS) influences the quality and uptake of ANC services.^24^ In Uganda, facilities that offered routine antenatal ultrasound services reported a 32% increase in the first ANC attendance compared to other facilities.^25^ One solution in resource-limited settings may be task shifting. Training of doctors, nurses and midwives in POCUS is effective.^26,27^

Late presentation for ANC posed another challenge in the study population where the prevalence of HIV was (40.8%). Fortunately, screening and treatment at the antenatal clinic were successful in meeting the Joint United Nations Programme on HIV/AIDS (UNAIDS) target of ensuring that more than 95% of ANC attendees knew their HIV status and more than 95% of ANC attendees diagnosed with HIV received ART.^28^ However, this study did not examine the viral load of pregnant women on their first visits. Other possible factors for late ANC presentation such as partner support, perceptions of clinic services and health provider behaviour were not evaluated in the current study. Late presentation for ANC was highlighted in the Saving Mothers report as a modifiable risk factor for adverse perinatal and maternal outcomes. This study did not report on the outcome of the pregnancies of the participants. However, there is a need for greater community awareness of the benefits of early initiation of ANC services.

### Limitations

This study was conducted at a single primary health care facility in KwaZulu-Natal among women with low-risk pregnancies. The results cannot be extrapolated to other provinces or to settings that manage high-risk pregnancies. Screening for the hepatitis B virus was introduced after the study period and was not audited in this study. Only quantitative data were included, and factors for late ANC enrolled requires a qualitative inquiry.

## Conclusion

The standard of ANC services at midwife-run unit met national standards. However, low uptake of early ANC services is of concern, especially in high HIV-prevalence settings. Increased community awareness and training of primary health care providers in POCUS may improve early uptake of ANC services.

## References

[1] Lee BX, Kjaerulf F, Turner S, et al. Transforming our world: Implementing the 2030 agenda through sustainable development goal indicators. J Public Health Policy. 2016;37(Suppl. 1):13–31. 10.1057/s41271-016-0002-7 27638240

[2] WHO. WHO recommendations on antenatal care for a positive pregnancy experience. Geneva: World Health Organization; 2016. 28079998

[3] World Health Organization. Trends in maternal mortality 2000 to 2017: Estimates by WHO, UNICEF, UNFPA, World Bank Group and the United Nations Population Division: Executive summary. Contract No.: WHO/RHR/19.23. Geneva: World Health Organization; 2019.

[4] Ward ZJ, Atun R, King G, Dmello BS, Goldie SJ. Global maternal mortality projections by urban/rural location and education level: A simulation-based analysis. EClinicalMedicine. 2024;72:102653. 10.1016/j.eclinm.2024.102653 38800798 PMC11126824

[5] Warri D, George A. Perceptions of pregnant women of reasons for late initiation of antenatal care: A qualitative interview study. BMC Pregnancy Childbirth. 2020;20(1):70. 10.1186/s12884-020-2746-0 32013894 PMC6998826

[6] World Health Organization. WHO recommendations on antenatal care for a positive pregnancy experience: Summary: Highlights and key messages from the World Health Organization’s 2016 global recommendations for routine antenatal care. Geneva: World Health Organization; 2018.

[7] Carroli G, Rooney C, Villar J. How effective is antenatal care in preventing maternal mortality and serious morbidity? An overview of the evidence. Paediatr Perinat Epidemiol. 2001;15(Suppl. 1):1–42. 10.1046/j.1365-3016.2001.00001.x 11243499

[8] Schmidt CN, Butrick E, Musange S, Mulindahabi N, Walker D. Towards stronger antenatal care: Understanding predictors of late presentation to antenatal services and implications for obstetric risk management in Rwanda. PLoS One. 2021;16(8):e0256415. 10.1371/journal.pone.0256415 34432829 PMC8386859

[9] Kuhnt J, Vollmer S. Antenatal care services and its implications for vital and health outcomes of children: Evidence from 193 surveys in 69 low-income and middle-income countries. BMJ Open. 2017;7(11):e017122. 10.1136/bmjopen-2017-017122 PMC5695442 29146636

[10] Habte A, Tamene A, Melis T. Compliance towards WHO recommendations on antenatal care for a positive pregnancy experience: Timeliness and adequacy of antenatal care visit in sub-Saharan African countries: Evidence from the most recent standard Demographic Health Survey data. PLos One. 2024;19(1):e0294981. 10.1371/journal.pone.0294981 38271342 PMC10810464

[11] Alem AZ, Yeshaw Y, Liyew AM, et al. Timely initiation of antenatal care and its associated factors among pregnant women in sub-Saharan Africa: A multicountry analysis of Demographic and Health Surveys. PLoS One. 2022;17(1):e0262411. 10.1371/journal.pone.0262411 35007296 PMC8746770

[12] Boah M, Issah A-N, Yeboah D, Kpordoxah MR, Sira J. Association between compliance with the new WHO-recommended frequency and timing of antenatal care contacts and receiving quality antenatal care in Cameroon. Sage Open. 2022;12(3):21582440221117807. 10.1177/21582440221117807

[13] Nyando M, Makombe D, Mboma A, Mwakilama E, Nyirenda L. Perceptions of pregnant women on antenatal care visit during their first trimester at area 25 health center in Lilongwe, Malawi – A qualitative study. BMC Womens Health. 2023;23(1):646. 10.1186/s12905-023-02800-7 38049740 PMC10696857

[14] Haffejee F, Ghuman S, Reddy P, et al. Timing of first antenatal care attendance and associated factors among pregnant women in an obstetric health facility in eThekwini district, KwaZulu-Natal Province, South Africa. Afr J Phys Activ Health Sci. 2018;24(2):181–192.

[15] Owigho O, Isara A. Women’s perception of quality and utilization of antenatal care and delivery services in Oshimili South Local Government Area of Delta State, Nigeria. J Commun Med Prim Health Care. 2022;34(1):81–98. 10.4314/jcmphc.v34i1.6

[16] Hlongwane TM, Bozkurt B, Barreix MC, et al. Implementing antenatal care recommendations, South Africa. Bull World Health Organ. 2021;99(3):220–227. 10.2471/BLT.20.278945 33716344 PMC7941100

[17] Ndlovu N, Gray A, Blose N, Mokganya M. Health and related indicators, 2023. S Afr Health Rev. 2023;2023(1):172–239. 10.61473/001c.122768

[18] National Department of Health. Saving mothers. Executive summary 2020–2024. Pretoria: South African National Department of Health; 2024.

[19] Patience NT, Sibiya MN, Gwele NS. Evidence of application of the basic antenatal care principles of good care and guidelines in pregnant women’s antenatal care records. Afr J Prim Health Care Fam Med. 2016;8(2):1–6. 10.4102/phcfm.v8i2.1016 PMC4913450 27380852

[20] Whelan R, Schaeffer L, Olson I, et al. Measurement of symphysis fundal height for gestational age estimation in low-to-middle-income countries: A systematic review and meta-analysis. PLoS One. 2022;17(8):e0272718. 10.1371/journal.pone.0272718 36007078 PMC9409500

[21] Okedo-Alex IN, Akamike IC, Ezeanosike OB, Uneke CJ. Determinants of antenatal care utilisation in sub-Saharan Africa: A systematic review. BMJ Open. 2019;9(10):e031890. 10.1136/bmjopen-2019-031890 PMC6797296 31594900

[22] Ngomane S, Mulaudzi FM. Indigenous beliefs and practices that influence the delayed attendance of antenatal clinics by women in the Bohlabelo district in Limpopo, South Africa. Midwifery. 2012;28(1):30–38. 10.1016/j.midw.2010.11.002 21146264

[23] World Health Organization. WHO antenatal care recommendations for a positive pregnancy experience. Maternal and fetal assessment update: Imaging ultrasound before 24 weeks of pregnancy. Geneva: World Health Organization; 2022. 35442602

[24] Abrokwa SK, Ruby LC, Heuvelings CC, Bélard S. Task shifting for point of care ultrasound in primary healthcare in low- and middle-income countries – A systematic review. eClinicalMedicine. 2022;45:101333. 10.1016/j.eclinm.2022.101333 35284806 PMC8904233

[25] Kawooya MG, Nathan RO, Swanson J, et al. Impact of introducing routine antenatal ultrasound services on reproductive health indicators in Mpigi District, Central Uganda. Ultrasound Q. 2015;31(4):285–289. 10.1097/RUQ.0000000000000142 26656991

[26] Mashamba T, Eyo A, Masilela S, Busakwe A, Towobola O. Limited obstetrics ultrasound in primary healthcare delivery; outputs for strategic considerations: A review of pilot study in Gauteng Province, South Africa. J Gynecol Obstet. 2022;10(2):52–59. 10.11648/j.jgo.20221002.11

[27] Bidner A, Bezak E, Parange N. Evaluation of antenatal Point-of-Care Ultrasound (PoCUS) training: A systematic review. Med Educ Online. 2022;27(1):2041366. 10.1080/10872981.2022.2041366 35382705 PMC8986272

[28] Woldesenbet S, Cheyip M, Lombard C, et al. Progress towards the UNAIDS 95-95-95 targets among pregnant women in South Africa: Results from the 2017 and 2019 national Antenatal HIV Sentinel Surveys. PLoS One. 2022;17(7):e0271564. 10.1371/journal.pone.0271564 35862306 PMC9302844

